# Global annual wetland dataset at 30 m with a fine classification system from 2000 to 2022

**DOI:** 10.1038/s41597-024-03143-0

**Published:** 2024-03-23

**Authors:** Xiao Zhang, Liangyun Liu, Tingting Zhao, Jinqing Wang, Wendi Liu, Xidong Chen

**Affiliations:** 1 International Research Center of Big Data for Sustainable Development Goals, Beijing, 100094 China; 2 Key Laboratory of Digital Earth Science, Aerospace Information Research Institute, Chinese Academy of Sciences, Beijing, 100094 China; 3 School of Electronic, Electrical and Communication Engineering, University of Chinese Academy of Sciences, Beijing, 100049 China; 4 College of Geomatics, Xi’an University of Science and Technology, Xi’an, 710054 China; 5 Future Urbanity & Sustainable Environment (FUSE) Lab, The University of Hong Kong, Hong Kong, 999007 China

**Keywords:** Hydrology, Limnology

## Abstract

Wetlands play a key role in maintaining ecological balance and climate regulation. However, due to the complex and variable spectral characteristics of wetlands, there are no publicly available global 30-meter time-series wetland dynamic datasets at present. In this study, we present novel global 30 m annual wetland maps (GWL_FCS30D) using time-series Landsat imagery on the Google Earth Engine platform, covering the period of 2000–2022 and containing eight wetland subcategories. Specifically, we make full use of our prior globally distributed wetland training sample pool, and adopt the local adaptive classification and spatiotemporal consistency checking algorithm to generate annual wetland maps. The GWL_FCS30D maps were found to achieve an overall accuracy and Kappa coefficient of 86.95 ± 0.44% and 0.822, respectively, in 2020, and show great temporal variability in the United States and the European Union. We expect the dataset would provide vital support for wetland ecosystems protection and sustainable development.

## Background & Summary

Wetlands are areas of land distinguished by the continual or occasional presence of water, where the water table is close to the surface of the soil, or where the land is covered by shallow water^1^. Regarded as one of the world’s most vulnerable and diverse ecosystems^2^, wetlands provide habitat for a vast range of species and are critical for regulating water resources^3^, storing carbon^4^, and moderating climate^5,6^. However, many wetlands around the world are threatened by factors such as human activity and climate change^7,8^. Wetland remote sensing mapping and monitoring provide not only real-time environmental data but also reveal changing trends and the health of wetland ecosystems^9–11^. As wetlands include a variety of surface covers such as marshes, swamps, bogs, fens, ponds, lakeshores, riverbanks, and estuaries^1,12^, their complicated spectral characteristics and heterogeneous spatiotemporal variabilities make mapping them quite difficult.

Over the past decades, global wetland remote sensing mapping has made some progress but mostly at coarse resolution^12–14^ mainly due to the limitations of huge computations and the limited free access of high-resolution satellite imagery. Many studies have emphasized that coarse wetland products cannot capture human-driven changes or small and fragmented wetlands^9,12,15^. Recently, breakthroughs in cloud computing and the improved accessibility of remote sensing archives have provided great opportunities for global wetland mapping. A series of high-resolution global thematic wetland products have been generated, including water bodies^16,17^, tidal flats^18–20^, mangrove forests^21–24^, and salt marshes^25–28^. Most of these products belong to coastal wetlands, and the high-resolution mapping of global inland wetlands (e.g., swamps and marshes) is sparse. Recently, Zhang, *et al*.^12^ combined multisourced remote sensing datasets to produce a global 30 m wetland dataset (named as: GWL_FCS30) containing five inland and three coastal wetland subcategories; however, it only covers a single year and cannot provide long-term wetland distribution information. Therefore, there are still no long-term global wetland distribution products with fine resolution, such as 30 m.

The major difficulty of wetland remote sensing mapping is how to obtain high-confidence training samples^29^. Previous studies have also highlighted that the confidence of training samples is the premise and key to high-precision land cover mapping^30,31^. In general, there are two channels to obtain the training samples: “visual interpretation” and “automatic generation”^12,32^. The former mainly relies on human prior knowledge and subjectivity to interpret the satellite imagery, and can achieve high-precision sample labeling under the premise of spending a large amount of manpower resources. In contrast, the latter makes full use of these published products to extract the confidence areas, and then uses the refinement method and automatic sampling to achieve the “automatic generation” of training samples^33–35^. Obviously, the “automatic generation” option can easily obtain the globally distributed training samples without any human participation. Therefore, it was also adopted to generate the GWL_FCS30 wetland dataset in 2020^12^.

Here, we generate novel global 30 m wetland annual maps with a fine classification system (GWL_FCS30D) from time-series Landsat imagery on the Google Earth Engine platform, which contains eight wetland subcategories—inland water, swamp, marsh, flooded flat, saline, mangrove, salt marsh, and tidal flat—and covers the time span of 2000–2022. The developed dataset will be the first global 30 m fine wetland annual maps from 2000 to 2022, which can provide strong support for understanding the spatial distributions and long time-series change of various wetland subcategories all over the world. The GWL_FCS30D dataset will also help to better understand and manage wetlands, thereby achieving sustainable natural resource management and environmental conservation goals.

## Methods

**Fig. 1:**
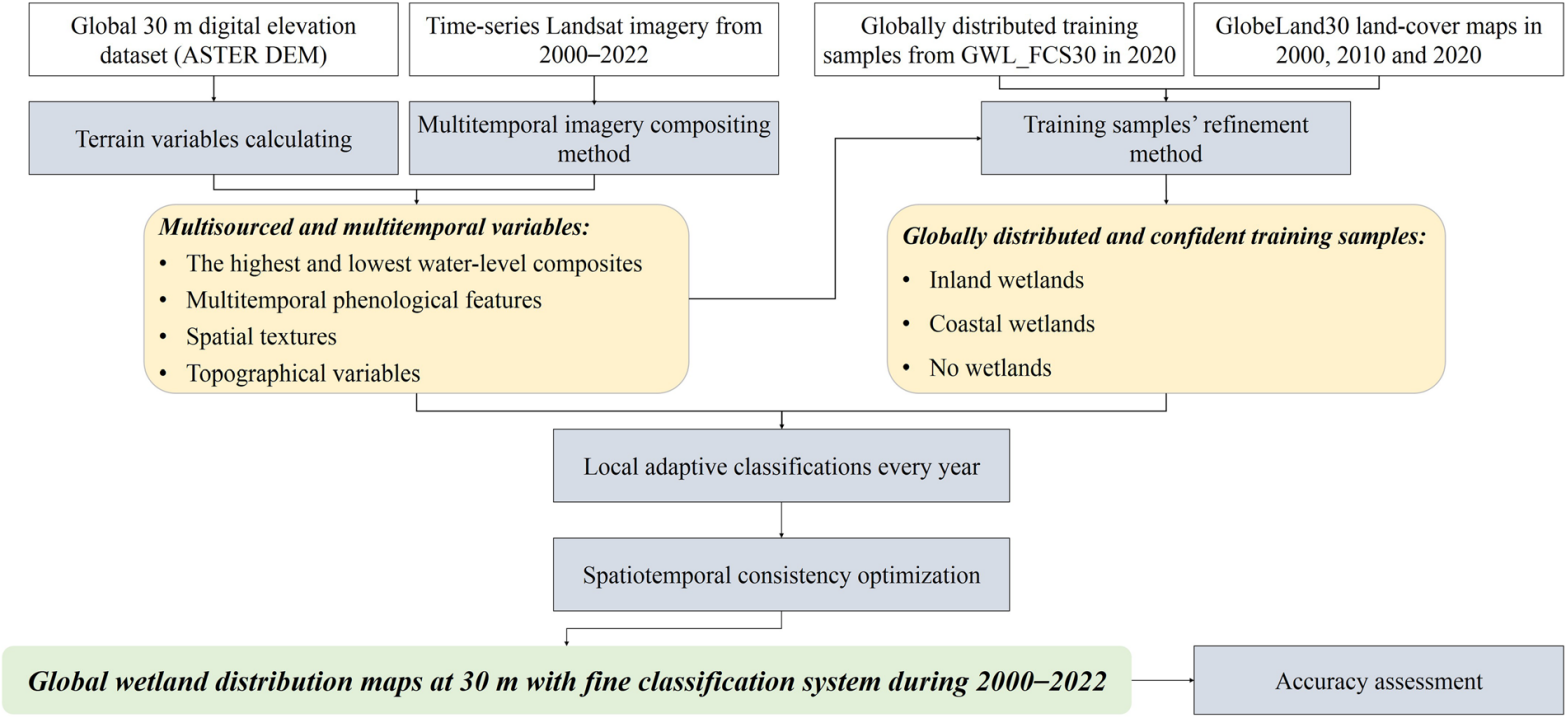
Flowchart of how to generate the global 30 m wetland annual maps from multisourced remote sensing imagery.

Figure 1 illustrates a flowchart of the developed method in mapping the global wetlands from time-series Landsat imagery. It contains four major parts: compositing multisourced and multitemporal training variables from time-series Landsat imagery, generating globally distributed and confident training samples over the whole period, the annual wetland remote sensing mapping and optimization algorithm, and generating the global 30 m wetland annual maps during 2000–2022 and analyzing their accuracy metrics. Details of each procedure are explained in the following sections.

### Remote sensing datasets

Landsat satellites, with their long-term and continual data recording, 30-m spatial resolution, 16-day revisit cycle, open data policy, and global coverage, provide great ability to capture the distributions and temporal changes of wetlands^36,37^. In this study, all Landsat imagery during 2000–2022, archived on the Google Earth Engine cloud-computing platform with < 80% cloud percentage, are used to generate the multitemporal and various water-level composites. To minimize the effects of atmosphere, clouds, snow, and ice, each Landsat image is first atmospherically corrected to the surface reflectance using the official algorithms of LEDAPS (Landsat Ecosystem Disturbance Adaptive Processing System)^38^ and LaSRC (Landsat Surface Reflectance Code)^39^; these “low-quality” pixels (e.g., cloud, shadow as well as ice and snow) are then masked based on the CFmask (C Function of Mask) algorithm, which was validated to achieve a high accuracy of 96.4%^40,41^ and thus adopted by the United States Geological Survey as an official algorithm.

The spatial distribution of wetlands is closely related to topography, that is, wetlands are easily formed in low-lying areas^12^. To characterize the topographical variability at 30 m, the ASTER Global Digital Elevation Model dataset, which is a globally comprehensive and highly detailed elevation dataset and undergoes regular updates and refinements to enhance its accuracy and reliability^42^, is used to capture the elevation features and corresponding slope and aspect using the topographical calculation functions of *ee.Terrain.slope()* and *ee.Terrain.aspect()* on the Google Earth Engine platform.

### Globally distributed and temporally stable training samples

Determining how to collect a globally distributed and confident training sample pool is the prerequisite for high-precision wetland mapping. Our previous works combined more than 10 global wetland-related products to generate a globally distributed training sample pool (denoted as GWL_TrainPool) containing 8 wetland subcategories (Table 1) and 4 non-wetlands (including forest/shrubland, grassland, cropland, and others), and then used it to develop the first global 30 m fine wetland map in 2020, achieving an overall accuracy of 86.44%^12^. Tables S1–1 & 2 also list the comparisons in the wetland classification system between this study and other previous wetland mappings or land-cover products, and it can be found that the fine classification system in GWL_FCS30D showed certain characteristics and advantages for global wetland mapping.

**Table 1:** Wetland classification system used in this study.

Name	Abbreviation	Description
Permanent water	PWT	Retaining water consistently throughout the year
Swamp	SWP	Inland natural wetlands mainly covered by tree vegetation (forest and shrub)
Marsh	MSH	Inland natural wetlands dominated by herbaceous vegetation
Flooded flat	FFT	Non-vegetated inland areas regularly inundated with water, typically during seasonal or periodic flooding events
Saline	SLE	High salt concentrations in the area’s water, soil, or vegetation
Mangrove	MGV	Salt-tolerant trees and shrubs that grow in brackish water
Salt marsh	SMH	Coastal wetland characterized by salt-tolerant vegetation, typically found in intertidal zones
Tidal flat	TFT	Muddy or rocky areas that are submerged during high tides and exposed during low tides

In this study, our key goal is making full use of GWL_TrainPool for supporting global annual wetland mapping during 2000–2022. It should be noted that the time representativeness of GWL_TrainPool is 2020, that is, it is inappropriate to directly use it to train the classification models in other years. Therefore, we must take some measures to refine GWL_TrainPool. Specifically, we firstly import multiple temporal GlobeLand30 land-cover datasets, which contained three time-steps of 2000, 2010, and 2020, and achieved an overall accuracy of 80.33%^43^, to refine these non-wetland samples in GWL_TrainPool. If a non-wetland sample was labeled as a water-body or wetland in any period of the GlobeLand30 datasets, it will be removed from the non-wetland sample pool. In other words, only temporally stable non-wetland samples are retained after the refinement.

In terms of vegetated wetlands (swamp, marsh, mangrove, and salt marsh) samples (Table 1), LandTrendr^44,45^ and VCT (Vegetation Change Tracker)^46^—two widely used land-cover change detection tools for monitoring vegetation disturbances (forest deforestation and vegetation degradation)—are applied to refine these vegetated wetland samples. Specifically, as LandTrendr and VCT are suitable for inter-annual land-cover changes, the inter-annual NDVI values of these vegetated wetlands in the 90th percentiles (explaining at next Section, minimizing the effects of residual cloudy) during 2000–2022 are input into the two models. If a vegetated wetland pixel is detected as having changed by either model, it is removed from GWL_TrainPool. In other words, only temporally stable vegetated wetland samples are retained after the change detection.

As tidal flats and flooded flats are only exposed at low tides, the inter-annual maximum LTideI values are analyzed to refine these flat samples. Specifically, our previous studies of mapping global tidal flats found that almost all tidal flats had positive LTideI indices in low tides^47^; that is, if any of the inter-annual maximum LTideI values of the flat sample are less than 0, then they will be discarded. As for how to refine the permanent water training samples, as permanent water in the JRC’s Global Surface Water (GSW) dataset was validated to achieve high user’s and producer’s accuracies of 99.1% and 99.7%^16^, respectively, the retained water samples are also permanent water bodies in the JRC-GSW dataset. The saline training samples are constrained through visual interpretation from time-series high-resolution images on the Google Earth platform, mainly because they are sparsely distributed and concentrated on several typical areas. Finally, the spatial distributions of sample size for these globally distributed samples is also given in the Figure S1, these sufficient and temporally stable training samples can effectively ensure the representativeness of regional wetlands.

### Compositing multisourced features

As the reflectance characteristics of wetlands are simultaneously influenced by water-level changes (e.g., tidal flats and flooded flats wetlands) and phenological variability (e.g., vegetated wetlands)^12^, the time-series Landsat imagery are composited into three types: the highest and lowest water-levels for identifying tide-sensitive subcategories (tidal flat, flooded flat, and permanent water body), multitemporal phenology (vegetated subcategories: swamp, marsh), and spatial textures. In terms of the highest and lowest water-levels, our previous study of mapping global tidal flats demonstrated that the maximum compositing method for the LTideI (low Tide index) and mNDWI (modified normalization difference water index) had a stronger ability to capture the lowest and highest tides^47^. Specifically, the LTideI index, optimized from the NDVI (Normalized Difference Vegetation index), made full use of the characteristics of tidal flats with high values in the near-infrared band and low values in other bands when comparing with sea water (Eq. (1)). Usually, the tidal flat achieved the positive value (>0) while ocean water was low negative value. The reason why LTideI used the maximum value of 4 spectral bands is that the visual bands of sea water were prone to negative value in high latitudes due to atmospheric correction error, the maximum measure can efficiently optimize this problem. Our previous study in global tidal flat mapping also demonstrated that it achieved stronger robustness and better performance than the NDVI index in splitting tidal flats with water body^47^. Then, the mNDWI was based on the spectral characteristic that water body had a higher reflectance values in visible bands than in NIR band, and usually achieved the positive value (>0) when covering with water, otherwise it would be lower than 0.

1 $$LTideI=\frac{{\rho }_{NIR}-\max \left({\rho }_{blue},{\rho }_{green},{\rho }_{red},{\rho }_{SWIR2}\right)}{{\rho }_{NIR}+\max \left({\rho }_{blue},{\rho }_{green},{\rho }_{red},{\rho }_{SWIR2}\right)+L}\times \left(1+L\right),$$

2 $$mNDWI=\frac{{\rho }_{green}-{\rho }_{NIR}}{{\rho }_{green}+{\rho }_{NIR}},$$

where *ρ*_*blue*_, *ρ*_*green*_, *ρ*_*red*_, *ρ*_*NIR*_, and *ρ*_*SWIR*2_ are the reflectance values of blue, green, red, and NIR (Near Infrared) and SWIR2 (Shortwave Infrared) bands in the Landsat imagery, and *L* is an adjustable term for improving the robustness of the LTideI index. The adjustable parameter *L* is selected as 0.1 based on the suggestion of Zhang, *et al*.^47^. The maximum compositing in LTideI is applied to represent the lowest tide, whereas the maximum mNDWI represents the highest tide. Specifically, the maximum compositing function of *qualityMosaic()*on the GEE platform was used to generate the maximum value of LTideI and mNDWI from the time-series Landsat imagery, and their corresponding reflectance bands (blue, green, red, NIR, SWIR1 and SWIR2 bands) were also composited. Namely, in the highest or lowest tide composites, the maximum value of mNDWI or LTideI as well as corresponding reflectance bands were generated. Thus, a total of 16 features are composited to represent the lowest and highest tides.

As for how to derive the phenological features from time-series Landsat observations, previous studies have categorized compositing methods into two groups: seasonal-based and percentile-based strategies^32,35,48^. Both strategy types share a similar ability to capture the phenology variability, but the former usually requires the seasonal calendar as prior knowledge. Thus, the percentile-based compositing strategy is more suitable for generating large-area phenology features and has also been widely used for global land-cover mapping^12,19,48^. The core of the percentile-based compositing strategy was to collect the magnitude of changing reflectance values instead of the time, that is, the phenological variations were reflected by different percentile values^49^. In this study, the intra-annual available Landsat data are composited into 10th, 30th, 50th, 70th, and 90th percentiles in blue, green, red, NIR, SWIR1, and SWIR2 bands, and NDVI, mNDWI, LSWI (Land Surface Water Index), and LTideI indices. NDVI and LSWI are introduced is to better capture the variabilities related to vegetation and water bodies. It should be emphasized that the maximum and minimum percentiles are generally ignored to minimize the residual effects of these “low-quality” pixels (e.g., cloud, shadow, ice, and snow).

3 $$NDVI=\frac{{\rho }_{NIR}-{\rho }_{red}}{{\rho }_{NIR}+{\rho }_{red}},LSWI=\frac{{\rho }_{NIR}-{\rho }_{SWIR2}}{{\rho }_{NIR}+{\rho }_{SWIR1}}$$

In addition to the multitemporal spectral features, the spatial textures are also collected to increase the separability between natural wetlands and some anthropogenic land-cover types, such as tidal flats and coastal aquaculture ponds^47^. Similar to our previous works in global tidal flat mapping^47^, the grey co-occurrence matrix algorithm is applied to the five NIR percentiles, and corresponding texture variables of entropy, contrast, variance, homogeneity, and correlation are generated to characterize the spatial variabilities at five percentiles. In short, after adding three topographical variables, a total of 94 multisourced variables are used for the subsequent classification modeling, including 16 spectral features for capturing the highest and lowest water-levels, 50 phenological features (5 percentiles × 10 variables), 25 texture variables, and 3 topographical variables.

### Wetland annual mapping using local adaptive classifications

Choosing an appropriate algorithm to map the global 30 m wetlands is also a critical step, and large-area land-cover classification and mapping often have two options: globally single modeling and local adaptive modelling. The former only builds a single classification model, which applies to the entire study area. In contrast, the latter splits the study area into multiply local areas and then builds the corresponding regional model in each local area. Some studies demonstrated that local adaptive modeling can achieve better performance than globally single modeling because the former can take into account the characteristics of each local area^33,50^. It should be noted that local adaptive modelling also requires a larger number of globally distributed training samples than globally single modeling. In this study, supported by the temporally stable and globally distributed training sample pool (GWL_TrainPoolstable), local adaptive modeling is adopted. We first divide the globe into 961 independent 5° × 5° geographical tiles, inherited from our previous works in global land-cover mapping^12,32,51^. It should be noted that the sample size in each 5° × 5° geographical tile ranges from approximately 2000 to 24000 because of their equal allocation sample distribution and the minimum sample size of 2000 for each land-cover type^12^. Then, the local classification models are trained using the regional training samples, and the local wetland maps are then generated from multisourced features. Meanwhile, it should be noted that the regional training samples came from the neighboring adjacent 3 × 3 geographical tiles, that is, the training samples in the central tile and its spatial neighboring adjacent tiles were combined to train the local adaptive classification model. The aims of this step are: (1) to improve the spatial continuous over the adjacent tiles^48^; and (2) increase training sample size especially for these sparse land-cover types. Similarly, our previous works in developing the GWL_FCS30 in 2020 also imported the spatial neighboring training samples^12^.

In terms of the specific classification algorithm, we directly use the random forest algorithm because of its high accuracy, resistance to overfitting, suitability for high-dimensional data, robustness, and ability to handle missing values and outliers^52–54^. We run the global annual wetland mapping on the Google Earth Engine platform using the function of *ee.Classifier.smileRandomForest()* with the default parameters and 100 decision trees.

### Temporal consistency optimization

We combine the temporally stable and globally training sample pool, multisource features, and local adaptive modeling to generate the global 30 m annual wetland maps. However, the influence of classification error and its accumulation cannot be ignored. To improve the temporal consistency of the time-series global wetland maps, the spatiotemporal consistency optimization method is employed (Eq. (4)):

4 $$P(x,y,t)=\frac{1}{N}{\sum }_{x{\prime} =x-{w}_{x}}^{x+{w}_{x}}\,{\sum }_{y{\prime} =y-{w}_{y}}^{y+{w}_{y}}\,{\sum }_{t{\prime} =t-{w}_{t}}^{t+{w}_{t}}I[L(x{\prime} ,y{\prime} ,t{\prime} )=L(x,y,t)]$$

where *P*(*x*, *y*, *t*) denotes the homogeneity probability at the spatial coordinates (*x, y*) and time point *t*, (*w*_*x*_, *w*_*y*_, *w*_*t*_) is the spatial and temporal window size for measuring the homogeneity, *L* represents the land-cover label, *N* is the size of spatiotemporal neighbor pixels and is equal to (*w*_*x*_, *w*_*y*_, *w*_*t*_), and *I()* is the indicator function and equals 1 when $L\left(x{\prime} ,y{\prime} ,t{\prime} \right)=L\left(x,y,t\right)$. In general, the spatial and temporal window size of (*w*_*x*_, *w*_*y*_, *w*_*t*_) are selected as the empirical value of 1, that is, the spatiotemporal nearest pixels (3 × 3 × 3) would participate in the optimization.

Specifically, we calculate the homogeneity probabilities for each pixel over the whole time-series $\left({P}_{x,y,2000},{P}_{x,y,2001},\ldots ,{P}_{x,y,2022}\right)$, and if the homogeneity probability at time-point *t* is less than 0.5 (empirical threshold suggested by the work of Li, *et al*.^55^), the land-cover type of this pixel at time-point *t* should be changed. However, some wetland subcategories are easily affected by water-level variations. For example, when rainfall is low in a given year, a large number of flooded flats and marshes will be exposed; otherwise, they are always covered by water bodies. Thus, some special change of wetland subcategories should be rejected during the post-processing step. We merge the permanent water, flooded flat, and marsh into one group, and the tidal flat, salt marsh, and permanent water (ocean) into another group, the swamp, mangrove forest and saline were remaining three independent groups. Namely, if the homogeneity probability is less than 0.5 and more than 1/3 in the same group, the land-cover change is rejected. It should be noted that the empirical threshold of 1/3 is used to solve the noise error in independent classification, that is, when the corresponding probability is less than 1/3, the land-cover label should be changed to the majority even in the same subcategory group.

## Data Records

**Fig. 2:**
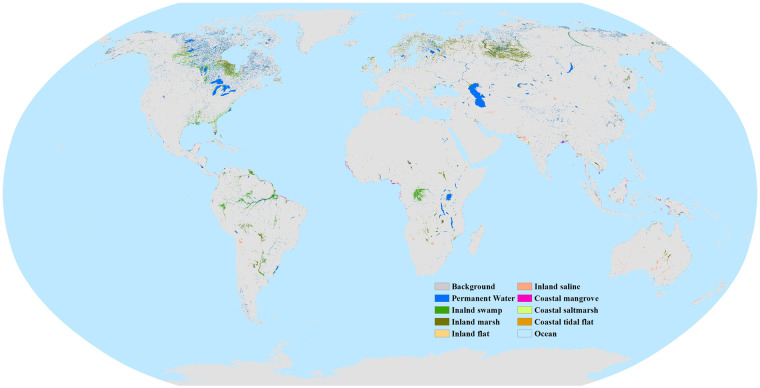
Spatial distribution of eight global wetland subcategories in 2022.

The developed global 30 m annual wetland maps with the fine classication system during 2000–2022 are freely shared via the Zenodo platform^56^. As saving the global 30 m wetland maps as a single file is too large, the shared global dataset is divided into 961 standard 5° × 5° geographical tiles with GeoTIFF format at the geographical projection and WGS84 coordinate system. Each tile contains 23 bands representing the year of wetland maps in 2000, 2001, …, 2021, and 2022, and is named as “GWL_FCS30D_20002022_E/W**N/S##”, where the “E/W**N/S##” specifies the longitude and latitude coordinates of the upper left corner of the grid. Then, the eight wetland subcategories, non-wetlands, and ocean are labeled with various values in each band, that is, the permanent water, swamp, marsh, flooded flat, saline, mangrove, salt marsh, and tidal flat are marked as 180, 181, 182, 183, 184, 185, 186, and 187, respectively; the non-wetland and ocean are labeled as 0 and 255, respectively. Figure 2 gives an overview of the eight global wetland subcategories in 2022 at 30 m spatial resolution. Overall, the GWL_FCS30 is spatially consistent with the actual global wetland patterns, namely, the inland wetlands (swamp, marsh, and permanent water) dominate the global wetlands and are mainly concentrated on the high latitudes of the Northern Hemisphere, rainforest areas, and areas adjacent to rivers and lakes.

## Technical Validation

### Accuracy assessment using global validation dataset

The wetlands usually reflected the variable spectral characteristics of water-level changes, and were the sparse land-cover types comparing with non-wetlands. How to ensure the sample size of wetland validation points and interpretation confidence of each validation point is the key for an accurate assessment. In this study, the stratified random sampling algorithm^57^ is applied to increase the sample size of these wetland points. Specifically, the total sample size *n* and for specific wetland subcategory *n*_*i*_ was determined by the Eq. (5),

5 $$n=\frac{{\left(\sum {W}_{i}\sqrt{{p}_{i}\left(1-{p}_{i}\right)}\right)}^{2}}{{\left(\frac{d}{t}\right)}^{2}+\sum {W}_{i}{p}_{i}\left(1-{p}_{i}\right)/N},\;{n}_{i}=n\times \frac{{W}_{i}\times {p}_{i}\left(1-{p}_{i}\right)}{\sum {W}_{i}\times {p}_{i}\left(1-{p}_{i}\right)}$$

where *N* is the number of pixel units in the study area; *t* defines the confidence interval (*t* = 1.96 for a 95% confidence interval, and *t* = 2.33 for a 97.5% confidence interval), and *d* denotes the desired half-width of the confidence interval; *W*_*i*_ is the weight distribution of class *i*; *p*_*i*_ is the producer’s accuracy. After the statistics, the preliminary sample size of *n* is determined as 24000.

**Fig. 3:**
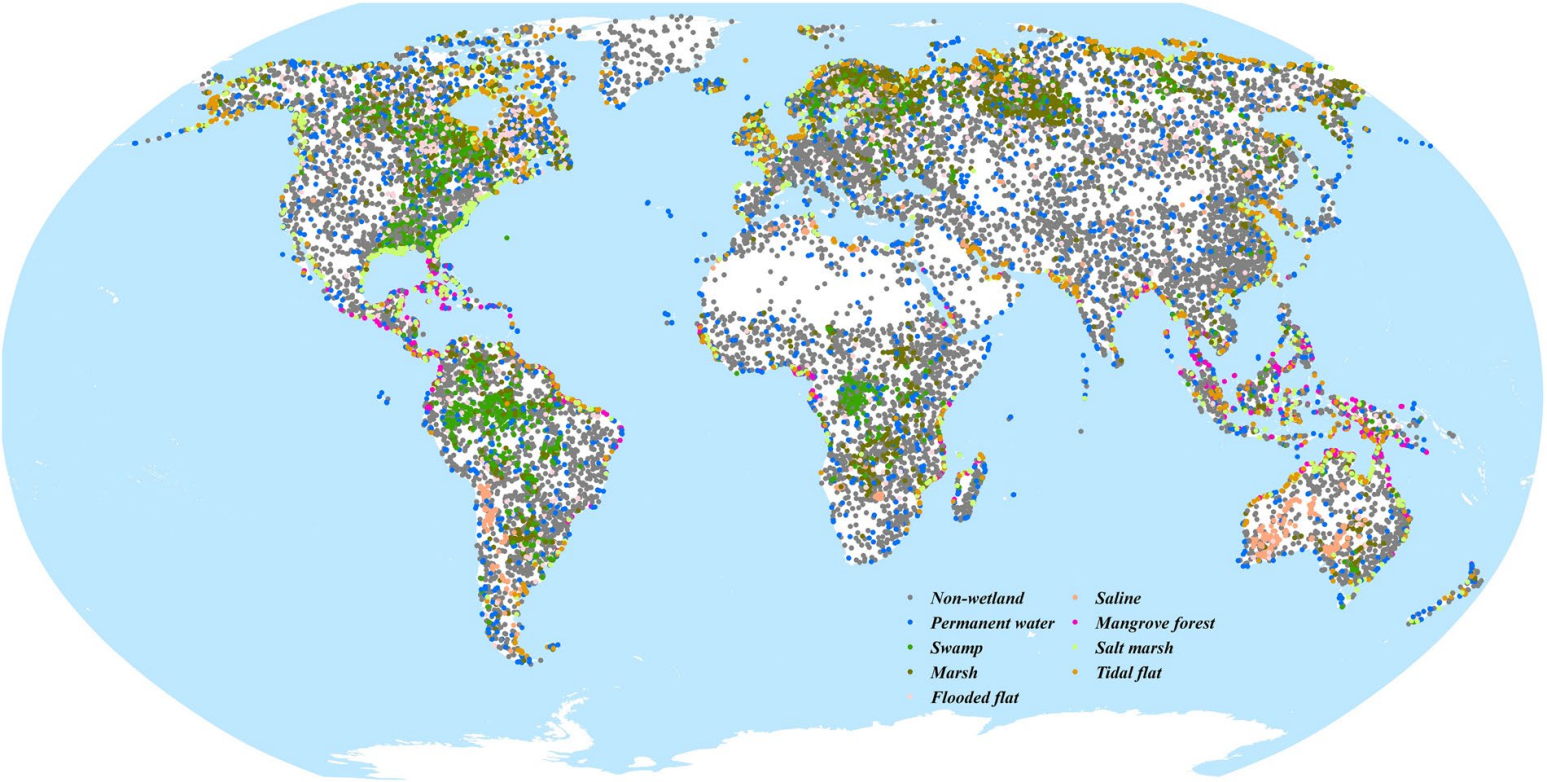
Spatial distribution of 22719 global validation points in 2020.

Then, in terms of how to determine the label of each validation point, we first make full use of the dense time-series Landsat and Sentinel-2 imagery to analyze the dynamic variability of each validation point and further combine the high-resolution imagery as auxiliary information to visually interpret the wetland and non-wetland status. In addition, to minimize the subjective of the validation samples, each validation point was independently interpreted by five experts, only the high agreement points are retained while these validation points with large differences among five experts were directly discarded. Afterwards, a total of 22719 validation points were collected for 2020, including 10952 non-wetland points and 11767 wetland points (7833 inland and 3934 coastal wetland points), as seen in Fig. 3. Overall, the distribution of wetland validation points basically reflected the actual global wetland distribution shown in Fig. 2.

The error matrix is one of the most widely used methods in accuracy assessment^57^. It generates four accuracy metrics: producer’s accuracy (P.A.), User’s accuracy (U.A.), overall accuracy (O.A.) and kappa coefficient (Kappa). Using the global wetland validation points and the error matrix calculation method, the error matrix of the developed global 30 m wetland maps in 2020 is depicted in Table 2. Our wetland maps showed good accuracy, as O.A. and Kappa reached 86.95 ± 0.44% and 0.822 in 2020, respectively. In terms of P.A. and U.A., we found that permanent water, mangrove, saline, and tidal flat had higher accuracies than that of the remaining wetland subcategories, mainly because they had unique spectral characteristics (permanent water, saline and tidal flat) or combined rich prior knowledge (mangrove). For example, tidal flats are only exposed for a short time and reflect water features at other times, and our previous work demonstrated the feasibility of LTideI index in capturing the exposed status of tidal flats^47^. In contrast, salt marsh had the lowest P.A. of 59.7 ± 2.51%, and it suffered evident confusions with tidal flats, mangrove, and coastal non-wetlands. The coexistence relationship of tidal flat, salt marsh and mangrove forest, as well as the complicated spectral characteristics of salt marshes, resulted in an omission error of 40.3%. Flooded flats and marshes also co-exist, and their distributions are usually affected by environmental factors (temperature and precipitation); thus, these environment-affected subcategory confusions decreased their P.A. and U.A. The PA of marsh and flooded flat was 64.13 ± 1.88% and 62.62 ± 3.77%, respectively.

**Table 2:** Error matrix of our wetland maps in 2020 using global validation points.

	NWT	PWT	SWP	MSH	FFT	SLE	MGV	SMH	TFT	Total	P.A. (%)
**NWT**	45.741	0.233	1.114	0.511	0.092	0.035	0.088	0.066	0.326	48.206	94.89(0.41)
**PWT**	0.313	7.39	0	0	0	0	0	0	0	7.703	95.94(0.92)
**SWP**	0.313	0.145	7.646	0.739	0	0.044	0.048	0	0.004	8.94	85.52(1.53)
**MSH**	0.079	0.524	2.874	7.034	0.198	0.079	0.035	0.088	0.057	10.969	64.13(1.88)
**FFT**	0.189	0	0.352	0.445	1.747	0.018	0.009	0.009	0.022	2.791	62.62(3.77)
**SLE**	0.11	0.026	0.013	0.075	0.185	3.658	0	0.004	0.004	4.076	89.74(1.96)
**MGV**	0.044	0.044	0.207	0.057	0.009	0	4.745	0.048	0.031	5.185	91.51(1.59)
**SMH**	0.357	0.233	0.172	0.242	0.374	0.009	0.546	3.847	0.665	6.444	59.70(2.51)
**TFT**	0.048	0.092	0.013	0.013	0.15	0.035	0.031	0.163	5.141	5.687	90.4(1.61)
**Total**	47.194	8.689	12.391	9.116	2.755	3.878	5.502	4.226	6.25		
**U.A. (%)**	96.92 (0.22)	85.06 (1.57)	61.71 (1.80)	77.16 (1.81)	63.42 (3.78)	94.32 (1.53)	86.24 (1.91)	91.04 (1.81)	82.25 (1.99)		
**O.A**.	86.95(0.44)										
**Kappa**	0.822										

Note: Refer to Table 1 for abbreviation definitions.

### Time-series accuracy metrics from third-party validation datasets

**Fig. 4:**
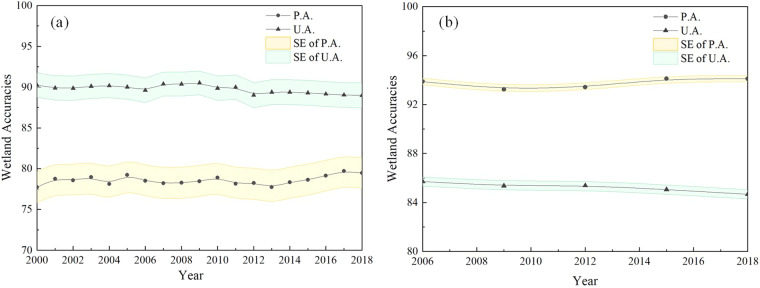
Time-series PA and UA wetland variability for our global 30 m wetland maps during 2000–2018 using the (**a**) LCMAP and (**b**) LUCAS validation datasets.

Collecting global time-series wetland validation points was a challenging and difficult task. Therefore, third-party time-series validation datasets LCMAP (Land Cover Monitoring, Assessment, and Projection) from the United States^58^ and LUCAS (Land Use/Cover Area frame Survey) from the European Union^59^ are used to objectively analyze the accuracy variability of our global wetland maps during 2000–2018. As wetland validation points are rare compared to other non-wetland points, only the P.A. and U.A. of wetlands and their standard error are presented in Fig. 4. Overall, our wetland maps showed good temporal stability in terms of P.A. and U.A. using the LCMAP and LUCAS datasets, reaching mean values of 78.32% and 93.52%, and 89.95% and 86.32%, for the two validation datasets, respectively. Specifically, for the LCMAP dataset, our wetland maps achieved higher U.A. (88.98%–90.52%) than the P.A. (77.73%–79.70%) values, which meant that our wetland maps suffered a higher omission error and a lower commission error. As for the LUCAS dataset, our maps achieved a higher P.A. of 93.24%–94.12% but a lower U.A. of 84.67%–85.71%, that is, the commission error was higher in LUCAS dataset of the European Union.

### Qualitative analysis of some typical areas

**Fig. 5:**
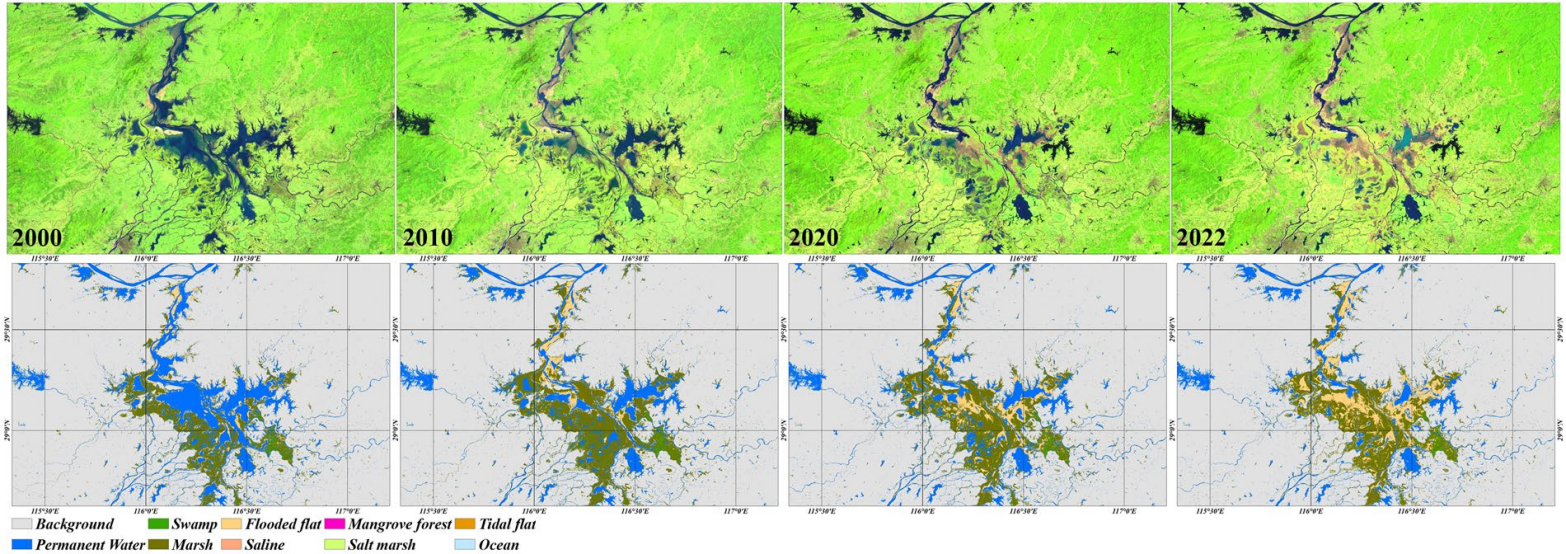
Spatial distribution of our time-series global wetland maps in Poyang Lake, China. The low-water-level composites are provided by corresponding Landsat imagery in the upper row using the SWIR1, NIR, and red false-color compositing.

To intuitively understand the performance of our time-series global wetland maps, a typical inland wetland case in Poyang Lake, China, in which the frequent changes in subcategories are affected by water-level and environmental factors, is illustrated in Fig. 5. Obviously, our wetland maps accurately capture the spatial distributions of four wetland subcategories (permanent water, swamp, marsh, and flooded flat), and four of them revealed various wetland distribution patterns in 2000, 2010, 2020 and 2022. For example, the permanent water area reached its minimum and maximum values in 2000 and 2022, respectively, with the main reasons being as follows: (1) the density of satellite observations affected the wetland remote sensing mapping, that is, the lowest water-level situation was easier to be captured from dense observations. (2) Environmental factors (temperature and precipitation) greatly influenced their distributions. For example, in 2022, the high temperature and low precipitation in the Poyang Lake area caused a large area of lake flats (flooded flats) to be exposed. In summary, our time-series wetland maps showed great ability to capture changes in these subcategories.

**Fig. 6:**
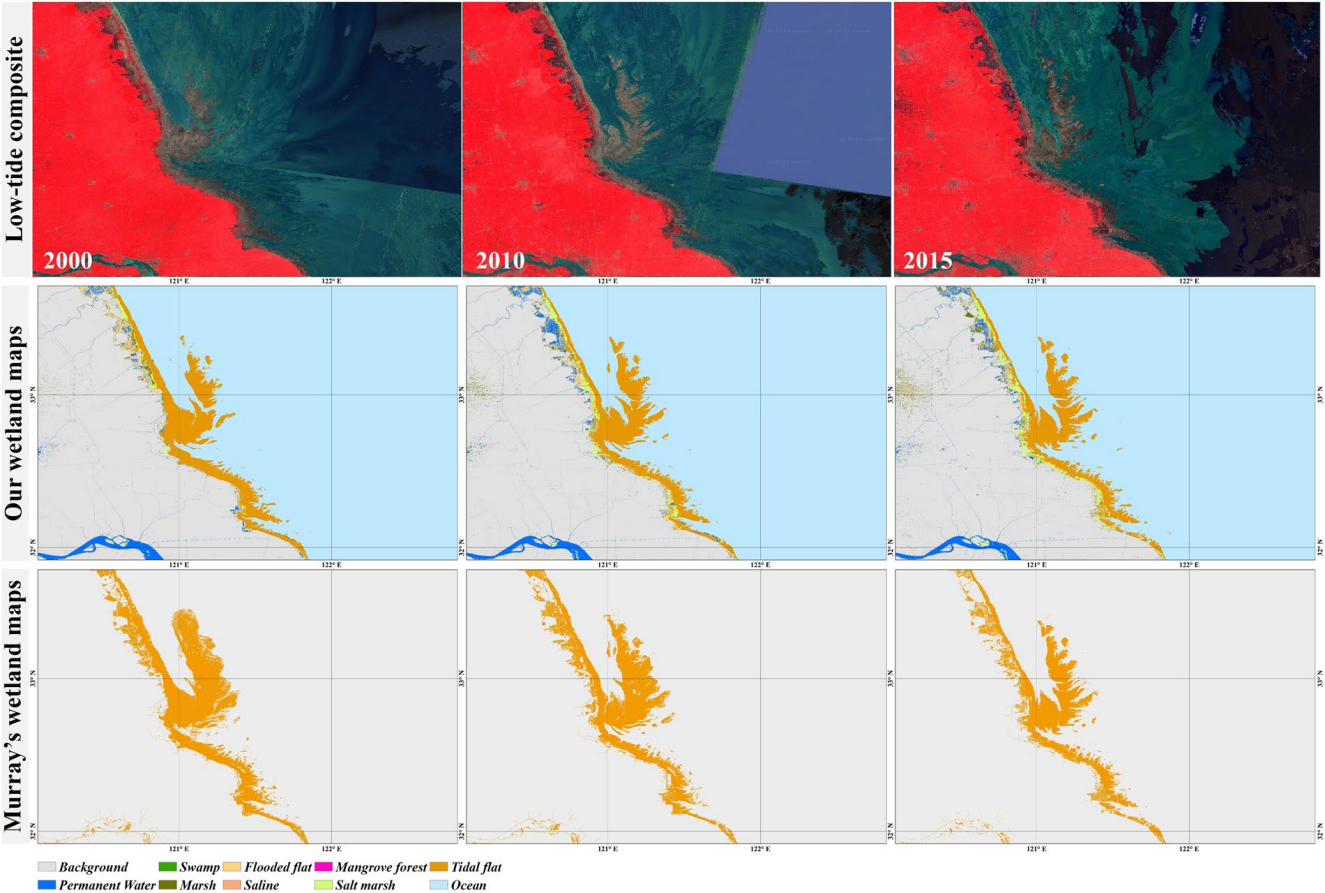
Comparison between our wetland maps and Murray’s global tidal flat products in Yancheng, Jiangsu province, during 2000–2015. Lowest tide composites are also given using false-color compositing (NIR, red, and green).

In terms of coastal wetlands, several 30 m global time-series mangrove and tidal flat products have been developed over the past few years^18,21–24^. We performed a cross-comparison between our wetland maps and other existing products in Yancheng, Jiangsu province, which contains one of the largest tidal flats in the world (Fig. 6). Overall, a higher consistency between the two datasets was found in 2015 than in the other two periods, and Murray’s tidal flat products evidently overestimated the tidal flat area in 2000 and 2010 according to the low-tide composites. In addition, our wetland maps had better performance in distinguishing tidal flats and other subcategories (e.g., coastal ponds and salt marshes), which share similar spectral characteristics and temporal variability, and were wrongly identified as tidal flats in Murray’s products. Previous studies also explained that Murray’s tidal flat products suffered from overestimation problems and misclassified some aquaculture ponds as tidal flats^47,60^. In addition, our wetland maps also present the distributions and temporal changes of salt marshes over the period of 2000–2015. Specifically, as there is a coexistence relationship between salt marshes and tidal flats, salt marshes were distributed on the edge of the tidal flats in our wetland maps, and showed a trend of area expansion during 2000–2015. Actually, the phenomenon was consistent with the actual situation, that is, salt marshes in the region have increased significantly over the past decade due to invasive alien species^61^.

**Fig. 7:**
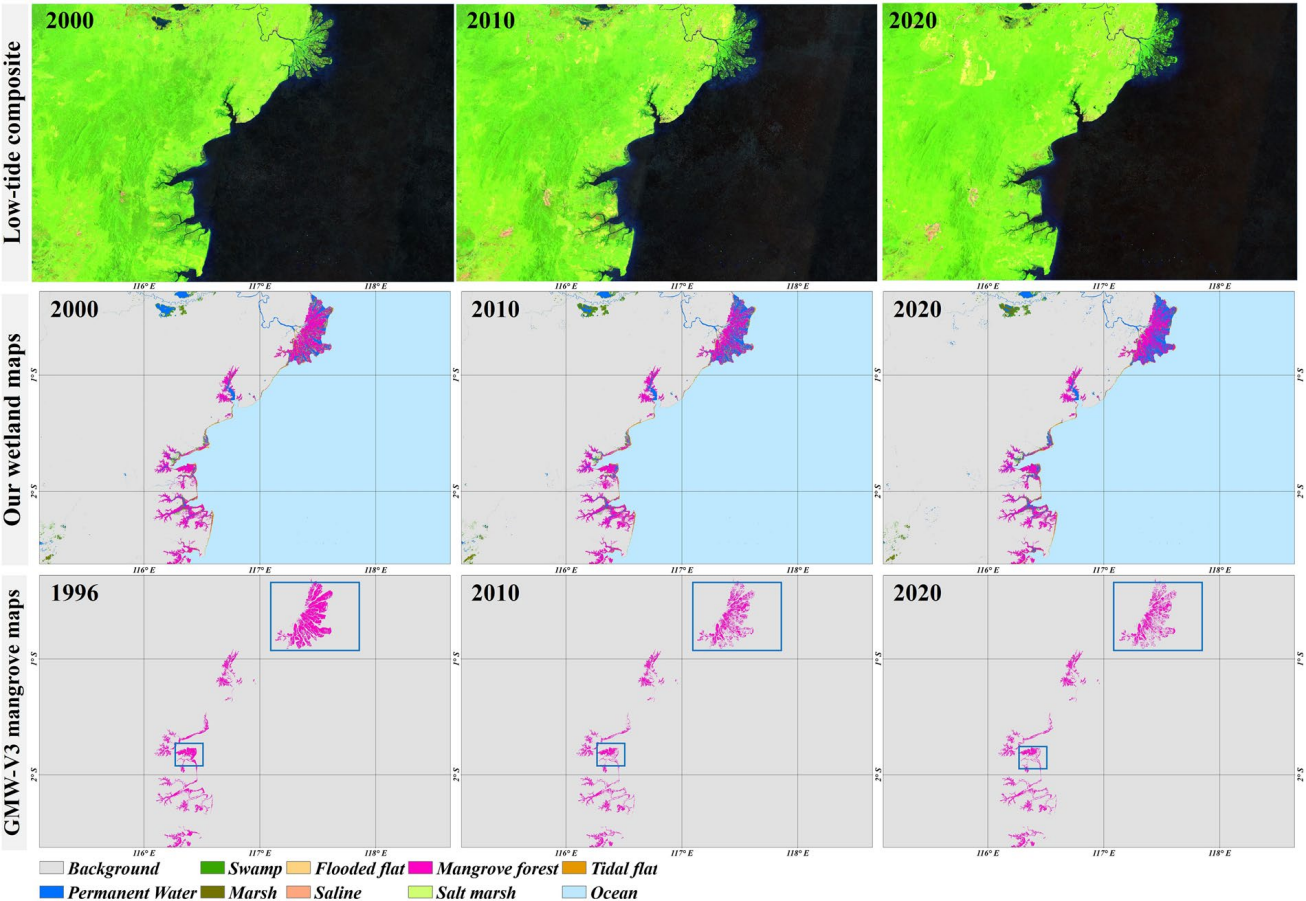
Comparison between our wetland maps with GMW-V3 products during 2000–2020 in Kalimantan Island, Indonesia. False-color composited Landsat imagery is also presented to capture the low tides.

Figure 7 provides another cross-comparison with the GMW-V3 (Global Mangrove Watch Project Version 3.0)^21^ dataset for Kalimantan Island, Indonesia, which contains the largest mangrove area of the world. It should be noted that the GMW-V3, developed from L-band Synthetic Aperture Radar imagery and covering the period of 1996–2020, has been proven to be the time-series mangrove monitoring product with the highest accuracy and performance currently, with an overall accuracy of 93.1% and a Kappa coefficient of 0.861^21^. In this comparison, we found that there was great consistency between the two wetland maps in the spatial patterns and temporal variability of mangrove: mangrove forest was distributed along the coastlines, most of which was concentrated in river estuary areas (e.g., Mahakam River’s estuary, the big blue rectangle in Figure 7). Meanwhile, in terms of temporal variability, the mangrove degradations, which were mainly reclaimed as coastal breeding ponds in the two blue rectangles of Figure 7, were accurately captured by our wetland maps and the GMW-V3 products. Thus, our time-series wetland maps also achieved reliable performance in capturing the spatial distributions and temporal changes of mangrove.

## Usage Notes

Our annual global wetland maps from 2000 to 2022 reveal the spatiotemporal distributions of eight wetland subcategories at a resolution of 30 m from the perspective of remote sensing observation, and can provide vital support in climate change analysis, carbon emission estimation, and regulating water resources^3^. To ensure the confidence of wetland mapping, we combined a temporally stable and globally distributed training sample pool, multisourced remote sensing features, a local adaptive modeling strategy, and spatiotemporal consistency optimization. The accuracy assessment and qualitative comparisons with existing products indicated that our wetland maps can capture the actual patterns and temporal changes of wetlands according to intra-annual Landsat imagery.

We still need to emphasize several points about our global wetland maps. First, as the generated wetland distributions came from remote sensing observations, the density of Landsat observations would affect their spatial patterns. For example, the area of flooded flats in Poyang Lake (Fig. 5) in 2000 is obviously less than that in later years, as there were insufficient Landsat satellite data during the low-water period in 2000. As there was no long time-span global DEM dataset on the GEE platform, the single-temporal ASTER DEM dataset around 2000 was used, that is, the topography changes over the period of 2000–2022 was ignored. Second, although we used globally distributed training samples and local adaptive modeling to ensure the mapping accuracy of wetlands, the problems of omission and commission errors due to complicated spatial and spectral characteristics of some wetland subcategories (marsh, flooded flat and salt marsh) still cannot be ignored, all of them suffered relatively low producer’s accuracy lower than 70% in Table 2. For example, the salt marsh had complicated spectral characteristics, and was susceptible to spectral mixing with tidal flats and mangrove forests. Third, we took a series of measures to ensure quality of the globally distributed and temporally stable training samples, however, it is impossible to filter all wetland changes due to their complicated spectra and temporal variations. For example, the changes within the vegetated wetlands still need to be strengthen. Thus, the future work will continue to optimize the quality of training samples by adding more prior knowledge and products or multisourced satellite imagery (e.g., Sentinel-1, JAXA SAR, and Sentinel-2 observations).

### Supplementary information

Supplement material

## Data Availability

The time-series Landsat analysis and wetland remote sensing mapping were programed on the Google Earth Engine platform with JavaScript, and the corresponding codes can be freely visited on the Zenodo platform^56^ and GitHub (https://github.com/zhangxiaoradi/CodeRepository/blob/main/GWL_FCS30D_Code).
