# Supplementary material for: Global annual wetland dataset at 30 m with a fine classification system from 2000 to 2022
Source: Sci Data. 2024 Mar 23;11:310. doi: 10.1038/s41597-024-03143-0 (PMC10960823; doi:10.1038/s41597-024-03143-0)
Supplement: Supplementary file 1 — Supplement material [file 41597_2024_3143_MOESM1_ESM.docx]

**Supplement materials**

# Methods

Table S1-1 and S1-2 lists the differences in wetland classification system among GWL_FCS30D, NWI and China’s wetland maps, as well as several typical global land-cover products. Overall, the GWL_FCS30D has great advantages over the global land-cover (GLC) products in diversity of wetlands (Table S1-2), and single wetland in the GLC products is divided into 4 inland and 3 coastal wetland categories. Then, in terms of the comparisons between GWL_FCS30D, China’s wetland maps ([Gong et al., 2010](#_ENREF_1)) and National Wetland Inventory products (NWI) ([Wilen and Bates, 1995](#_ENREF_2)), the national wetland products mainly focus on the permanent water body. For example, [Gong et al. (2010)](#_ENREF_1) further divided the permanent water into rivers, lakes, ponds/reservoirs, artificial river channels.

**Table S1-1**. The comparisons in classification system between GWL_FCS30D, China’s wetland in Gong et al., (2010) and NWI products.

| **GWL_FCS30D** | | **China’s wetland in Gong et al., (2010)** | | **NWI** |
| --- | --- | --- | --- | --- |
| **Category I** | **Category II** | **Category I** | **Category II** | **Category** |
| Inland wetland | Permanent water | Inland wetland | Rivers | Freshwater- Forested and Shrub wetland |
|  | Swamp |  | Flood plain wetlands | Freshwater Emergent wetland |
|  | Marsh |  | Lakes | Freshwater pond |
|  | Flooded flat |  | Inland marshes | Estuarine and Marine wetland |
|  | Saline | Coastal wetland | Tide zone/shallow beach | Riverine |
| Coastal wetland | Mangrove |  | Marine marshes | Lakes |
|  | Salt marsh |  | Estuarine water | Estuarine and Marine Deepwater |
|  | Tidal flat |  | Estuarine deltas | Other Freshwater wetland |
|  |  |  | Lagoons |  |
|  |  | Artificial wetland | Reservoirs/ponds |  |
|  |  |  | Artificial river channels |  |
|  |  |  | Seawater fish farms |  |
|  |  |  | Rice and paddy fields |  |
|  |  |  | Recreational water bodies |  |
|  |  |  | Other |  |

**Table S1-2.** The wetland details in several typical global land cover products.

| **GLC_FCS30, GlobeLand30, FROM_GLC** | **CCI_LC** | **MCD12Q1** | **ESA WorldCover** |
| --- | --- | --- | --- |
| Wetland | Tree cover, flooded, fresh or brakish water | Permanent wetlands | Herbaceous wetland |
| Water body | Tree cover, flooded, saline water | Water Bodies | Mangroves |
|  | Shrub or herbaceous cover, flooded, fresh/saline/brakish water |  | Permanent water bodies |
|  | Water bodies |  |  |

Figure S1 illustrates the spatial distribution of training sample size at each 1°×1°geographical grid cell. Overall, the training samples were mainly concentrated on the coastal areas in which contained all coastal wetland samples, as well as part of inland wetland and non-wetland samples. On the contrary, arid and semi-arid areas are relatively sparse because there are no wetlands here. From the perspective of total sample size, our globally distributed training samples size reaches the order of tens of millions or more, which effectively guarantees the representativeness of regional wetlands.


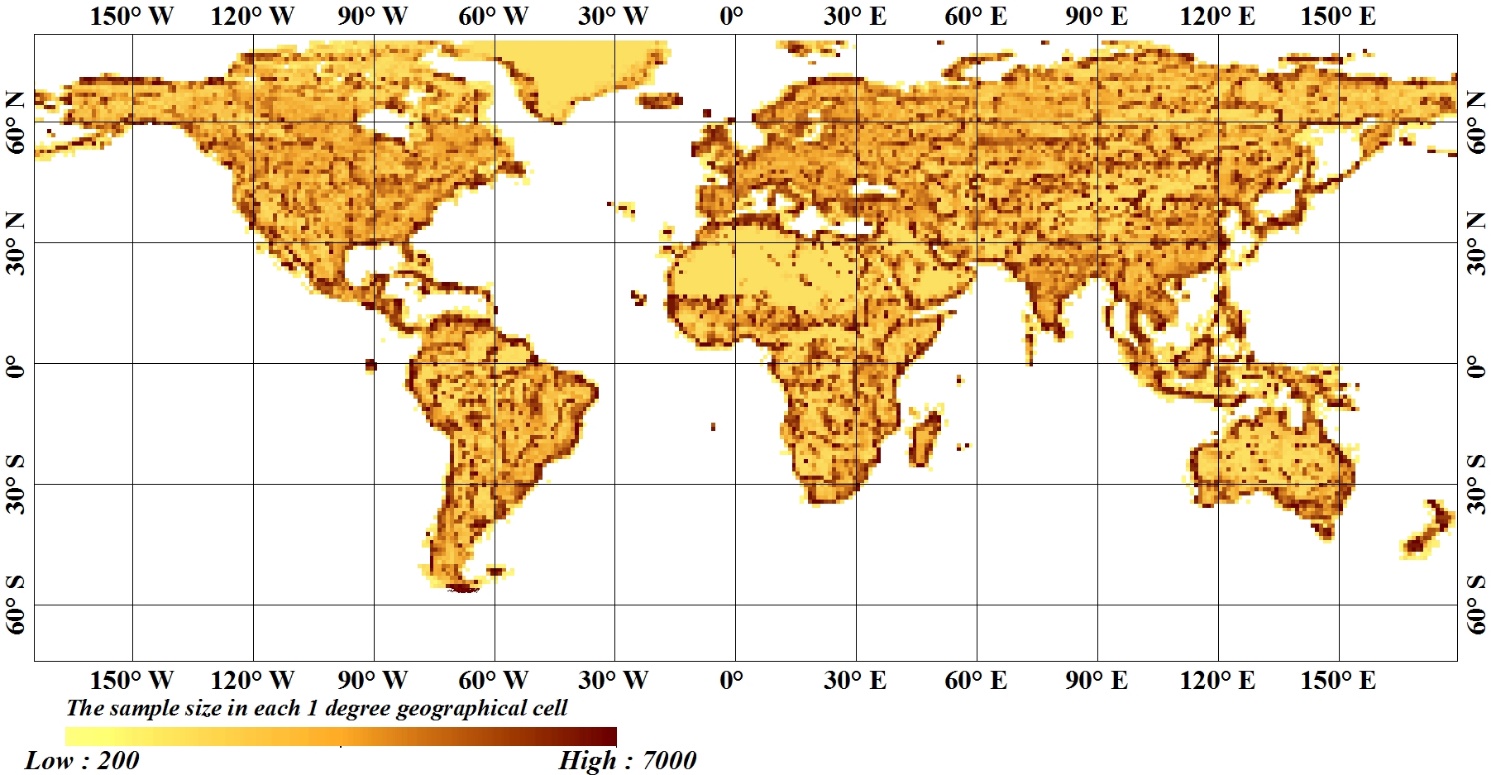


**Figure S1**. The spatial distribution of training sample size at each 1°×1°geographical grid cell.

# References

Gong, P., Niu, Z., Cheng, X., Zhao, K., Zhou, D., Guo, J., Liang, L., Wang, X., Li, D., Huang, H., Wang, Y., Wang, K., Li, W., Wang, X., Ying, Q., Yang, Z., Ye, Y., Li, Z., Zhuang, D., Chi, Y., Zhou, H., and Yan, J.: China’s wetland change (1990–2000) determined by remote sensing, Science China Earth Sciences, 53, 1036-1042, <https://doi.org/10.1007/s11430-010-4002-3>, 2010.

Wilen, B. O. and Bates, M.: The US fish and wildlife service’s national wetlands inventory project. In: Classification and inventory of the world’s wetlands, Springer, 1995.
